# Inverted Encoding Models Reconstruct an Arbitrary Model Response, Not the Stimulus

**DOI:** 10.1523/ENEURO.0363-18.2019

**Published:** 2019-03-26

**Authors:** Justin L. Gardner, Taosheng Liu

**Affiliations:** 1 Department of Psychology, Stanford University, Stanford, CA 94305; 2 Department of Psychology, Michigan State University, East Lansing, MI 48824

**Keywords:** computation, feature, fMRI, representation, tuning, vision

## Abstract

Probing how large populations of neurons represent stimuli is key to understanding sensory representations as many stimulus characteristics can only be discerned from population activity and not from individual single-units. Recently, inverted encoding models have been used to produce channel response functions from large spatial-scale measurements of human brain activity that are reminiscent of single-unit tuning functions and have been proposed to assay “population-level stimulus representations” (Sprague et al., 2018a). However, these channel response functions do not assay population tuning. We show by derivation that the channel response function is only determined up to an invertible linear transform. Thus, these channel response functions are arbitrary, one of an infinite family and therefore not a unique description of population representation. Indeed, simulations demonstrate that bimodal, even random, channel basis functions can account perfectly well for population responses without any underlying neural response units that are so tuned. However, the approach can be salvaged by extending it to reconstruct the stimulus, not the assumed model. We show that when this is done, even using bimodal and random channel basis functions, a unimodal function peaking at the appropriate value of the stimulus is recovered which can be interpreted as a measure of population selectivity. More precisely, the recovered function signifies how likely any value of the stimulus is, given the observed population response. Whether an analysis is recovering the hypothetical responses of an arbitrary model rather than assessing the selectivity of population representations is not an issue unique to the inverted encoding model and human neuroscience, but a general problem that must be confronted as more complex analyses intervene between measurement of population activity and presentation of data.

## Significance Statement

We recently showed that inverted encoding models conflate signal-to-noise ratio with neural tuning width. Sprague and colleagues argued that despite this short falling, inverted encoding models “assay population-level stimulus representations.” However, we show that inverted encoding models reconstruct the model responses, not the stimulus. This is problematic because the model, as we derive here, is only determined up to a linear transform and thus the recovered model responses are only one of an infinite family of equivalent solutions. The approach thus fails to provide a unique assay of population representation. This problem can be circumvented by extending the approach to estimate the probability of different values of the stimulus, thus resulting in an interpretable assay of population representation.

## 

There is no cone type in the human retina that responds selectively and uniquely to the color chartreuse. Nor is there a cone type for fuchsia, indigo, ebony, crimson, azure, or cerulean. Not even for the three color primaries: red, green, and blue. Rather, the relative activity of just three different receptor types was hypothesized (Young, 1802), and later validated through color-matching experiments (Helmholtz, 1867), to give rise to the multitude of color sensations. This population code for color contrasts with a pure labeled line hypothesis in which each color sensation would be due to a single class of uniquely devoted neurons (Doetsch, 2000). Even for sensory structures like the olfactory system that maintain strictly segregated connectivity from odorant receptor types in the olfactory epithelium to glomeruli in the olfactory bulb, individual odorants can activate numerous different odorant receptors leading to combinatorial possibilities that allow discrimination of many tens of thousands of different compounds despite there being only a few hundred distinct odorant receptors in humans (Buck, 2004). These key findings in sensory physiology firmly place population coding, that is, the idea that for each distinct sensory percept there is some invariant spatiotemporal pattern of activity that can only be discerned from a population rather than a single neuron, as a fundamental concept of sensory representation.

**Figure 1.**
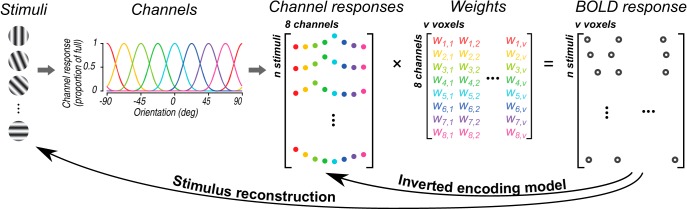
Overall schematic of the channel encoding model and its applications. A number of stimuli varying along a dimension of interest (in this case, orientation) are presented (“stimuli”) and neural responses are measured. The measured neural responses are assumed to reflect summed activity from a set of underlying mechanisms (“channels”), which are characterized by basis functions that resemble tuning curves of sensory neurons. Each channel’s response to each stimulus can be calculated based on the channel’s basis function (“channel responses”). These channel responses are multiplied by a weight matrix (“weights”) that reflects the relative contribution of each channel in each voxel (i.e., *w_i,j_* is the contribution of *i^th^* channel in *j^th^* voxel). The weighted sum of the channel responses produces the measured neural response (“BOLD response”). By calculating the weights and inverting the model on independent datasets, the inverted encoding model recovers a set of channel responses, whereas by taking into account the structure of the model, one can also reconstruct the stimuli that most likely generated the measured neural responses. To facilitate visualization, each channel and its associated responses and weights are depicted in a different color.

Recently, it has been proposed that an inverted encoding model approach to analysis of functional imaging data from human cortex can assay such “population-level stimulus representations” (Sprague et al., 2018a). However, here, we show that it is the model assumed in the analysis that is reconstructed, not the stimulus. Moreover, the model is arbitrary in that it is only specified to within a linear transform and thus unsuitable for assaying population representation. Typically, encoding models (Naselaris et al., 2011; Serences and Saproo, 2012) are used as lower-dimensional representations of complex sensory stimuli whose responses are then used as linear predictors of cortical responses. For example, a channel encoding model (Brouwer and Heeger, 2009) is one in which a continuous variable like color (Brouwer and Heeger, 2009, 2013; Yu and Shim, 2017), orientation (Brouwer and Heeger, 2011; Ho et al., 2012; Scolari et al., 2012; Ester et al., 2013, 2015, 2016; Garcia et al., 2013; Byers and Serences, 2014; Chong et al., 2016; Bullock et al., 2017; Yu and Shim, 2017; Liu et al., 2018; Lorenc et al., 2018), direction of motion (Saproo and Serences, 2014; Chen et al., 2015), or spatial location (Sprague and Serences, 2013; Sprague et al., 2014, 2016, 2018b; Samaha et al., 2016; Vo et al., 2017) is conceived of exciting several channels with different selectivity for the variable. To take a specific example, hypothetical orientation channels (channel basis functions) with different preferred orientations but identical bandwidths (typically a sinusoidal function raised to an exponent) are created (Fig. 1). The selectivity of the orientation channels are meant to mimic the known selectivity of individual primary visual cortex neurons (Campbell et al., 1968; Rose and Blakemore, 1974; Watkins and Berkley, 1974; Gardner et al., 1999; Ringach et al., 2002; Finn et al., 2007). For each oriented stimulus that is presented, one can calculate how the hypothetical channels would respond. Across many presentations of different stimuli, a matrix of channel responses is constructed and regression coefficients (weights) can be calculated that best predict each voxels’ response in a functional magnetic resonance imaging experiment. After fitting these regression coefficients on a training dataset, predicted channel responses can be computed by inverting the procedure for some left-out dataset, by multiplying the pseudo-inverse of the voxel regression coefficients with the observed voxel responses. If there is reliable selectivity in the population response for the stimulus variable, the resulting predicted channel responses will exhibit a tuned profile that approximates the channel basis functions built into the analysis.

This approach has been called an inverted encoding model (Sprague et al., 2018a) to emphasize that it is an extension to the more typical approach which uses an encoding model to predict BOLD responses (Dumoulin and Wandell, 2008; Kay et al., 2008; Brouwer and Heeger, 2009) without then inverting the procedure to estimate the model responses. The tuned profiles that inverted encoding models produce have been used to characterize population stimulus representations across different task contexts such as during working memory (Ester et al., 2013, 2015; Foster et al., 2016; Sprague et al., 2016; Yu and Shim, 2017; Lorenc et al., 2018) or comparisons across different allocations of attention (Scolari et al., 2012; Garcia et al., 2013; Sprague and Serences, 2013; Ester et al., 2016). Simulations show that these predicted channel responses can index neural tuning in that the widths of the functions change with the width of the underlying selectivity of neurons in the population. However, the predicted channel response functions also change width as a function of the overall signal-to-noise ratio of the measurement, thus conflating neural selectivity with noise (Liu et al., 2018; Sprague et al., 2018a).

If these predicted model responses are to be taken as measures of population stimulus representations, it raises the question as to what exactly a “stimulus representation” is. A long tradition in physiology has measured neural responses as sensory stimuli are systematically varied to assess the relationship between neural response and stimulus properties. Perhaps the most fundamental relationship is that of the receptive field (Hartline, 1938), which is now commonly used in a stimulus space-referred (rather than the original sensory-organ referred) fashion, as when it describes the location within the visual field from which a response can be elicited. As physiologists discovered more complex response properties of single neurons to stimulus features such as orientation (Hubel and Wiesel, 1959, 1962), it became common to characterize neural tuning functions. That is, the response as measured as a function of parametric variation of a stimulus, such as orientation (Campbell et al., 1968; Rose and Blakemore, 1974; Watkins and Berkley, 1974; Gardner et al., 1999; Ringach et al., 2002; Finn et al., 2007). Tuning functions have been used to characterize the stimulus representation not only by the firing rate of single-units, but also by other neural measures such as membrane potentials (Finn et al., 2007; Priebe and Ferster, 2012), EEG potentials (Maffei and Campbell, 1970; Regan and Regan, 1987; Baker et al., 2011), reflectance changes from intrinsic signals (Grinvald et al., 1986; Swindale et al., 2003), fluorescence signals from voltage-sensitive dyes (Benucci et al., 2007; Chen et al., 2012), and calcium-imaging measurements (Ohki et al., 2005). Even for BOLD activity averaged across a visual area, parametric sensitivity to the strength of a visual stimulus can be assessed by plotting response magnitude as a function of stimulus properties like contrast (Tootell and Taylor, 1995; Boynton et al., 1996, 1999; Tootell et al., 1998; Logothetis et al., 2001; Avidan et al., 2002; Olman et al., 2004; Gardner et al., 2005; Pestilli et al., 2011) or motion coherence (Rees, 2000; Braddick et al., 2001; Costagli et al., 2014; Birman and Gardner, 2018), which are expected to result in monotonic increases in response of all neurons in a population. Typical for all of these characterizations of stimulus representation is that they report a measurement of neural activity as a stimulus property is systematically varied. Some tuning functions may be derived through a number of analytic steps, such as when computing a tuning function (DeAngelis et al., 1993; Gardner et al., 1999) from a reverse-correlation mapped receptive field profile (Jones and Palmer, 1987) or when Fourier components are computed in a frequency-tagged EEG measurement (Regan and Regan, 1987; Baker et al., 2011; Tsai et al., 2012; Verghese et al., 2012). Nonetheless, the interpretation is straight-forward: the representation characterizes neural response as a function of stimulus variation.

While inverted encoding models can generate a predicted channel response function visually similar to these classically measured tuning functions, the ordinate of the graph is no longer a direct measurement of neural activity. Indeed, a rather odd feature of the literature using inverted encoding model is that there is a lack of consensus over what units to label the ordinate with. It has been alternately labeled as arbitrary units (Brouwer and Heeger, 2011; Ho et al., 2012; Ester et al., 2013; Garcia et al., 2013; Byers and Serences, 2014; Chong et al., 2016), without any specified units (Sprague and Serences, 2013), normalized units (Saproo and Serences, 2014) or in the units of the measurement, for example, as the percentage signal change of BOLD response (Brouwer et al., 2015), or the power of an EEG measurement (Samaha et al., 2016; Bullock et al., 2017), or normalized BOLD (Chen et al., 2015) or BOLD *z* score (Sprague et al., 2014, 2016, 2018b; Ester et al., 2015; Vo et al., 2017), or relative magnitude (Scolari et al., 2012; Chong et al., 2016; Yu and Shim, 2017). The units of the ordinate are arbitrary in the sense that they can be manipulated by simply changing the maximum response of the modeled channels. Typically set to a unit value, if instead, the maximum channel response is set to two, in the ideal case of no noise in response or measurement, the inverted encoding model will produce predicted channel response functions with doubled height. Making the channel response functions to have a maximum response of forty-two will produce predicted values that will scale accordingly, without any change in the underlying measured responses. Thus, despite being linearly weighted responses, because the maximum channel response can be arbitrarily scaled, the predicted channel response no longer reflects the units of the measurement. Instead, this arbitrary scaling of the ordinate with model assumptions can be avoided by simply plotting the ordinate in proportion or percentage of the full model response (Liu et al., 2018). Because the inverted encoding model is simply a linear regression that attempts to predict channel responses from BOLD responses (Fig. 1), in the limit of no noise, the predicted channel response functions should approach the full amplitude of the model basis functions. Put another way, imagine an encoding model in which one predicts BOLD response magnitude from the age of the subject. If one were to invert this encoding model, then BOLD responses would be used to predict age, and the ordinate would be in units of what is being predicted, years of age, rather than in the units of the predictor, percentage signal change. Viewed as producing proportion of the full model response, the predicted channel response function lies in stark contrast to other tuning functions in which the ordinate is a measurement of neural activity. Thus, the output of the inverted encoding model, i.e., the channel response function, is not a measured response against different stimulus values. Instead, it is the predicted response of a hypothetical modeled channel.

**Figure 2.**
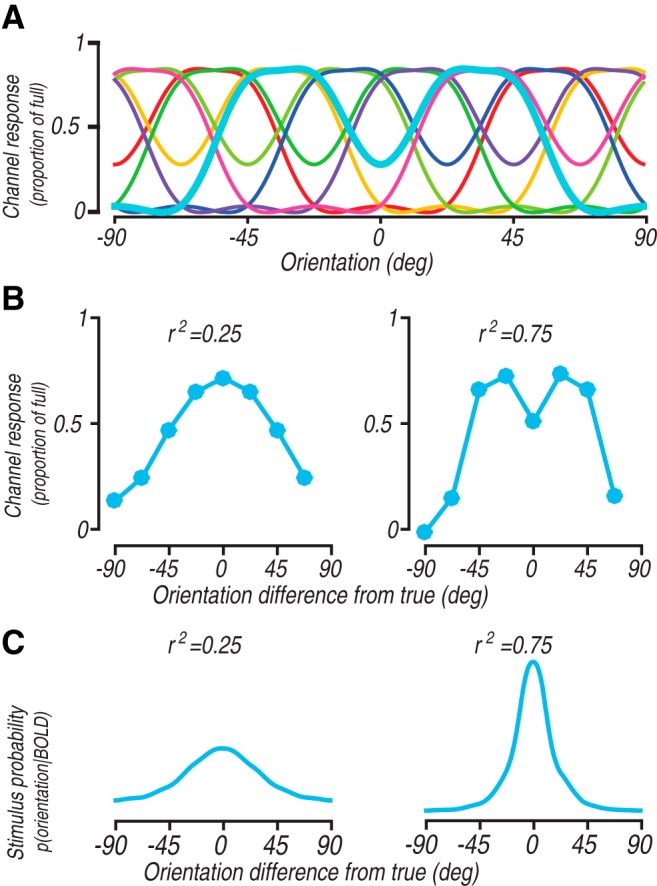
Simulation results with a bimodal basis function. ***A***, Depiction of eight channel basis functions, each one with two peaks positioned ∼67° apart. To facilitate visualization, the center channel (cyan) is plotted in a thicker line. The channels are obtained by multiplying the original channels (Fig. 1) with a matrix that transforms the unimodal to a bimodal shape. ***B***, Channel response functions derived by the inverted encoding model at high noise (left panel) and low noise (right panel) levels. ***C***, Posterior probability of the stimulus derived by the Bayesian approach at high noise (left panel) and low noise (right panel) levels.

To better explicate the distinction between a classical tuning function and the predicted channel response function, it is instructive to consider a, seemingly, extreme case of poor model specification. We therefore built and tested a channel encoding model on a synthetic data set using published techniques (Liu et al., 2018), except that we changed the channel basis function to have a bimodal shape (Fig. 2*A*). We ran the channel encoding model on simulated data, using procedures identical to those previously reported (Liu et al., 2018). Briefly, the model contained 100 voxels, where each voxel was assumed to contain a random proportion of neurons sampled from a bank of identical, orientation tuned neurons with uniformly distributed orientation preference. Neural tuning functions were circular Gaussians as implemented by von Mises functions. The random proportions in each voxel constitute a weight vector that specifies the contribution of each neuron to the voxel’s response. When presented with a stimulus, the response of each neuron was calculated using its neural tuning function, and the response of each voxel was calculated as a weighted sum of the neuronal response according to the voxel’s weight vector. Independent Gaussian noise with standard deviation systematically varied to simulate different amounts of noise was added to this response to yield a final response of each voxel. We then simulated an experiment in which eight evenly spaced orientation stimuli were each presented 27 times (Liu et al., 2018) to generate BOLD responses for each trial.

Despite the fact that simulated responses were generated by neurons with unimodal tuning functions, the inverted encoding model with bimodal channels can produce a bimodal channel response function. For example, with a unimodal neural tuning width of 40° (half-width at half-height of the von Mises) and at low noise level (high r^2^), channel response function had a bimodal shape (Fig 2*B*, right panel), which is expected given that we have shown that the predicted channel response function converges to the channel basis function at low noise level (Liu et al., 2018). We also note that at a higher noise level (low r^2^), the channel response appeared unimodal (Fig. 2*B*, left panel). Critically, the predicted channel response function does not reflect the underlying neural tuning of the simulated data. The bimodal shape of the predicted channel response function is entirely a consequence of the choice of encoding model basis functions, not of any particular consequence of the modeled responses. This is troubling for an interpretation of the channel response function as a measure of population stimulus representation, because it simply recapitulates the model assumptions, in this case of bimodality, rather than any intrinsic property of the simulated data. While the simulations show that a bimodal channel response function emerges as noise is reduced, it would clearly be a mistake to use this analysis and conclude that the population stimulus representation has changed from a unimodal to a bimodal function across these two simulated conditions.

**Figure 3.**
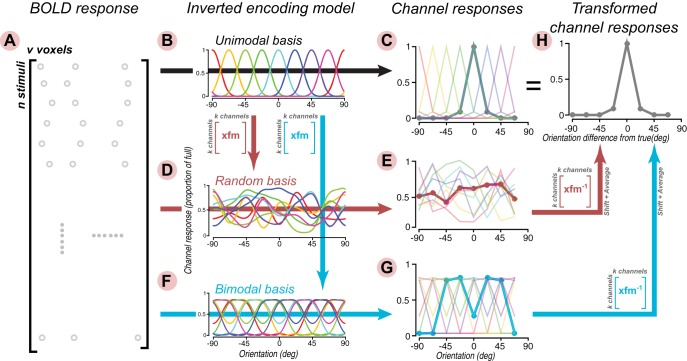
Illustration of the behavior of the inverted encoding model under transformed channel basis functions. The simulated BOLD responses (***A***) are generated as before, assuming a set of unimodal neuronal tuning functions. In the first case, standard channel basis functions (depicted in ***B***) are used to estimate the weights and invert the model (depicted by the horizontal arrow), which gives rise to a set of channel response functions (***C***). Here, we depicted both individual channel responses (colored lines) and the shifted and averaged channel response (thick gray line); the latter is typically reported in the literature, and duplicated in ***H***. In the second case, the standard channel basis functions are multiplied by a transformation matrix filled with random numbers (depicted in the red matrix) to generate a set of new basis functions (***D***). After model inversion, individual and averaged channel responses are seemingly random (***E***). In the third case, a set of bimodal basis functions (***F***; same as Fig. 2*A*) were obtained by multiplying the standard basis functions with an appropriate transform (depicted in the blue matrix), which yielded bimodal channel response functions after model inversion (***G***). When the individual channel responses in ***E***, ***G*** are multiplied by the inverse of their respective transforms, shifted, and averaged, an identical channel response is obtained as in the standard unimodal case (***H***). To facilitate visualization, these simulations were conducted assuming zero noise. The same results also hold under non-zero noise conditions.

While one might think that the issue is one of poor model specification that could be resolved through appropriate usage of model comparison statistics, it is not. In fact, the amount of variance accounted for by the encoding model using the typical unimodal functions (Fig. 1) and the bimodal functions is identical. Indeed, the bimodal encoding model, though obviously “wrong,” was constructed as a linear transform of the “right” unimodal model and thus is mathematically interchangeable (Fig. 3*B*,*F*). More specifically, the unimodal and bimodal channel basis functions were defined as follows:

(1) $$R_{1}=SC_{1}\text{and}R_{2}=SC_{2}\text{where}C_{2}=C_{1}P$$

Where the Rs are n × k (n = number of trials, k = number of channels) matrices of channel response functions (Fig. 1, channel responses). The stimuli S are projected onto the channel basis functions C. S is a n x s (s = number of different stimulus types) stimulus matrix with zeros everywhere except for a one in each row at the appropriate column to indicate which stimulus type was presented during that trial. The Cs are s × k matrices which contain channel basis functions in the columns evaluated at each of the stimulus values. The subscripts indicate the unimodal (1) and bimodal (2) channel basis functions. P is an invertible channel conversion matrix (k × k) which we have designed to convert the unimodal channel basis functions into bimodal functions. Thus, the channel response matrices for the unimodal and bimodal basis functions are related as follows:

(2) $$R_{2}=SC_{2}=SC_{1}P=R_{1}P$$

By construction then the unimodal and bimodal channel basis functions span the same linear subspace and therefore both encoding models account for the same amount of variance. In fact, the weight matrices for the two models are related by a linear transform. To see this, consider the equations for how the encoding model accounts for BOLD responses (Brouwer and Heeger, 2009; Serences and Saproo, 2012; Liu et al., 2018):

(3) $$B=R_{1}W_{1}+\eta \text{and}B=R_{2}W_{2}+\eta$$

Where B is a n × v (v = number of voxels) matrix of BOLD responses for all trials, the Ws are k × v weight matrices and η is zero mean Gaussian noise. The weight matrices can be estimated using least squares estimation from a training set of BOLD data $B_{T}$ (Brouwer and Heeger, 2009; Serences and Saproo, 2012; Liu et al., 2018):

(4) $${\hat{W}}_{1}={\left(R_{1}^{T}R_{1}\right)}^{-1}R_{1}^{T}B_{T}\text{and}{\hat{W}}_{2}={\left(R_{2}^{T}R_{2}\right)}^{-1}R_{2}^{T}B_{T}$$

Where the superscript T indicates transpose and –1 indicates inverse. The relationship between the estimated weights for the model with the bimodal basis functions, ${\hat{W}}_{2}$, and the unimodal functions, ${\hat{W}}_{1}$ can be derived as follows:

$${\hat{W}}_{2}={\left(R_{2}^{T}R_{2}\right)}^{-1}R_{2}^{T}B_{T}$$

$${\hat{W}}_{2}={{{((R}_{1}P)}^{T}R_{1}P)}^{-1}{\left(R_{1}P\right)}^{T}B_{T}$$

by substitution of Equation 2

$${\hat{W}}_{2}={\left(P^{T}R_{1}^{T}R_{1}P\right)}^{-1}P^{T}R_{1}^{T}B_{T}$$

by expansion of transpose

$${\hat{W}}_{2}={P^{-1}{{{\left(R_{1}^{T}R_{1}\right)}^{-1}P}^{T}}^{-1}P}^{T}R_{1}^{T}B_{T}$$

by expansion of inverse

$${\hat{W}}_{2}={P^{-1}{\left(R_{1}^{T}R_{1}\right)}^{-1}R}_{1}^{T}B_{T}$$

multiplication by inverse is identity

(5) $${\hat{W}}_{2}=P^{-1}{\hat{W}}_{1}$$

by substitution of Equation 4

Thus, in sum, the unimodal and bimodal channel basis functions span the same subspace, account for the same amount of variance in the encoding model, and the estimated weight matrices are related by a linear transform.

In fact, both models will produce identical predictions for stimulus test values that were never even used to train the models. Let ${\hat{B}}_{1,LO}$ and ${\hat{B}}_{2,LO}$ be the predicted BOLD responses for the unimodal and bimodal models, respectively, for test stimuli $S_{LO}$ that were left out of the training set. Note that $S_{LO}$will have dimensions n_lo_ × s_lo_ for the number of left out stimuli and the number of types of left out stimuli. The channel basis functions $C_{1,LO}$and $C_{2,LO}$ will have dimensions s_lo_ × k because they are evaluated at each of the s_lo_ left out stimulus values. By Equations 1, 3, the predicted BOLD responses for the unimodal and bimodal models are as follows:

(6) $${\hat{B}}_{1,LO}=S_{LO}C_{1,LO}{\hat{W}}_{1}\text{and}{\hat{B}}_{2,LO}=S_{LO}C_{2,LO}{\hat{W}}_{2}$$

We can show that ${\hat{B}}_{1,LO}$ and ${\hat{B}}_{2,LO}$ are equal as follows:

$${\hat{B}}_{2,LO}=S_{LO}C_{2,LO}{\hat{W}}_{2}$$

$${\hat{B}}_{2,LO}=S_{LO}C_{1,LO}P{\hat{W}}_{2}$$

by substitution of Equation 1

$${\hat{B}}_{2,LO}=S_{LO}C_{1,LO}PP^{-1}{\hat{W}}_{1}$$

by substitution of Equation 5

$${\hat{B}}_{2,LO}=S_{LO}C_{1,LO}{\hat{W}}_{1}$$

multiplication by inverse is identity

$${\hat{B}}_{2,LO}={\hat{B}}_{1,LO}$$

by substitution of Equation 6

Thus, both encoding models produce exactly the same predictions for BOLD responses even for stimulus test values for which the models were not trained on.

Not only are the unimodal and bimodal encoding models interchangeable and produce identical predictions, the inverted encoding models result in estimated channel response functions that are a linear transform of each other. Consider the way in which channel response functions are estimated from a held-out validation BOLD data set, $B_{V}$ (Brouwer and Heeger, 2009; Serences and Saproo, 2012; Liu et al., 2018):

(7) $${{\hat{R}}_{1}=B}_{V}{\hat{W}}_{1}^{T}{\left({\hat{W}}_{1}{\hat{W}}_{1}^{T}\right)}^{-1}\text{and}{{\hat{R}}_{2}=B}_{V}{\hat{W}}_{2}^{T}{\left({\hat{W}}_{2}{\hat{W}}_{2}^{T}\right)}^{-1}$$

The relationship between the estimated channel response functions using the inverted encoding model with unimodal, ${\hat{R}}_{1}$, and bimodal, ${\hat{R}}_{2}$, channel basis functions can be derived as follows:

$${{\hat{R}}_{2}=B}_{V}{\hat{W}}_{2}^{T}{\left({\hat{W}}_{2}{\hat{W}}_{2}^{T}\right)}^{-1}$$

$${{\hat{R}}_{2}=B}_{V}{\left(P^{-1}{\hat{W}}_{1}\right)}^{T}{\left(P^{-1}{\hat{W}}_{1}{\left(P^{-1}{\hat{W}}_{1}\right)}^{T}\right)}^{-1}$$

by substitution of Equation 5

$${{\hat{R}}_{2}=B}_{V}{\hat{W}}_{1}^{T}{P^{-1}}^{T}{\left(P^{-1}{\hat{W}}_{1}{\hat{W}}_{1}^{T}{P^{-1}}^{T}\right)}^{-1}$$

by expansion of transpose

$${{\hat{R}}_{2}=B}_{V}{\hat{W}}_{1}^{T}{P^{T}}^{-1}{\left(P^{-1}{\hat{W}}_{1}{\hat{W}}_{1}^{T}{P^{T}}^{-1}\right)}^{-1}$$

Interchange transpose and inverse

$${{\hat{R}}_{2}=B}_{V}{\hat{W}}_{1}^{T}{P^{T}}^{-1}P^{T}{\left({\hat{W}}_{1}{\hat{W}}_{1}^{T}\right)}^{-1}P$$

by expansion of inverse

$${{\hat{R}}_{2}=B}_{V}{\hat{W}}_{1}^{T}{\left({\hat{W}}_{1}{\hat{W}}_{1}^{T}\right)}^{-1}P$$

multiplication by inverse is identity

(8) $${\hat{R}}_{2}={\hat{R}}_{1}P$$

by substitution of Equation 7

Thus, one can take the reconstructed bimodal channel response functions from the inverted encoding model analysis and turn them back into unimodal channel response functions by multiplying them by the inverse of the linear transform used to create the bimodal channel basis functions (Fig. 3*G*,*H*).

As the recovered channel response functions from the inverted encoding model are only constrained up to an invertible linear transformation, the channel response functions can even be converted randomly. As long as the transformation to the random channel basis functions is an invertible transformation, the analysis will result in estimated channel response functions that can be converted through a linear transform back into the unimodal functions (Fig. 3*D*,*E*). Indeed, the channel response functions can be converted between any of the infinitely many equivalent channel response functions related by invertible transforms. In this sense, the particular choice of channel basis functions to display within these infinite possibilities is a completely arbitrary assumption of the analysis and cannot be interpreted as uniquely indicative of the population representation.

This problem of recapitulating the arbitrary model assumptions with an inverted encoding model can be circumvented by using a related Bayesian approach (van Bergen et al., 2015; van Bergen and Jehee, 2018) which computes the posterior probability of the stimulus given the measured responses. The Bayesian approach follows the same structure as an inverted encoding model analysis, but characterizes the residual variance as due to independent, identically distributed noise from the channels and independent and correlated components of voxel noise (for our voxel model we did not simulate correlated voxel noise so we did not fit this component). Having fit both the channel model and the noise, the probability of producing any particular response given a stimulus can be computed. Using Bayes’ rule and a uniform prior, the posterior probability of any stimulus given a particular response can then be computed. Using this approach with the exact same simulated data and bimodal encoding model, we found a posterior always centered at the actual stimulus orientation, with its spread reflecting the uncertainty (Fig. 2*C*). Similar behavior was observed over a range of combinations of parameters. This approach highlights a useful interpretation of these model responses. The posterior function represents what probability one could guess the stimulus orientation after having observed a BOLD response. The wider the function, the more uncertain the stimulus orientation is. Notably, the approach yields a unimodal posterior function regardless of whether the channel basis functions are unimodal (van Bergen et al., 2015; Liu et al., 2018) or bimodal as simulated here. This is a sensible outcome as it shows the peak probability at the actual stimulus orientation which decays uniformly around that orientation.

The reason for this striking difference in which the Bayesian approach produces a unimodal posterior and the inverted encoding model yields a bimodal channel response function is simply because the Bayesian approach aims at stimulus reconstruction rather than model reconstruction (Fig. 1). Given a neural response and a model for how that response could be generated, stimulus reconstruction attempts to determine what stimulus occurred (Stanley et al., 1999). To simplify the task, identification of the most likely stimulus among a finite number of possibilities (Kay et al., 2008) or classification into a number of discrete categories (Haxby et al., 2001; Kamitani and Tong, 2005) and/or the use of more simplified stimuli (Miyawaki et al., 2008) have all been used. There can be no claim about whether that representation of the stimulus is used in the brain, only that information is available in the measured responses that can be used to recreate the stimulus. Reconstruction, identification and classification have been used in many experiments to compare sensory responses under different cognitive states like attention (Kamitani and Tong, 2005, 2006; Jehee et al., 2011; Dobs et al., 2018) or working memory (Harrison and Tong, 2009), examine the influence of priors and expectancy (Kok et al., 2012, 2013; Vintch and Gardner, 2014) and a wide variety of other purposes. Channel encoding models have also been fruitfully used for stimulus reconstruction, for example by reconstructing color values that the model was never trained on (Brouwer and Heeger, 2009).

The inverted encoding model approach does not aim to reconstruct the stimulus, but rather aims to reconstruct an intermediate step of the analysis: the encoding model’s representation of the stimulus. The parameters of the tuning functions of different channels in the encoding model are often taken to mimic the selectivity of neurons or groups of neurons, yet the reconstructed channel response functions do not unambiguously reflect the tuning properties of these neurons (Liu et al., 2018). Therefore, the predicted channel response that the analysis recreates exists only as a theoretic construct; it is neither inherent in the stimulus nor in the population representation. As demonstrated above, a bimodal channel response can be reconstructed from a population representation that was built from unimodal representations of the stimulus. However, the Bayesian analysis, despite using the same bimodal encoding model, recovers a unimodal posterior because it aims to reconstruct the stimulus rather than the model. While channels for basic stimulus properties like color, orientation and spatial frequency can be informed by existing physiologic literature, model specification is less well constrained for more complex stimulus properties and the possibility of poor model specification giving rise to misleading results becomes more likely. To be clear, building encoding models based on well-understood tuning functions even with the ambiguities described here is not necessarily problematic as it can be a useful way to reduce the dimensionality of the stimulus space in a principled way. However, inverting the encoding model even for these cases where the single-unit tuning functions are well known, simply recapitulates the assumptions about the channel basis functions, such as their tuning width, and therefore does not provide a useful assay of population tuning. Thus, inverted encoding models produce a result that is not interpretable as a population nor a neural tuning function, but instead is an estimate of the arbitrary model basis function.

Rather than inverting the encoding model to display the fit to the intermediate model assumptions, examining the weights that are needed to explain population responses can be informative about the population representation. That is, encoding models without inversion, have often been used to understand population representations. For example, a Gabor wavelet model can be used to encode visual stimuli into spatially local filters with different orientation and spatial frequency selectivity meant to mimic the selectivity of primary visual cortex neurons (Kay et al., 2008). After fitting such a model, the location, orientation and spatial frequency selectivity can be determined for each voxel, allowing for retinotopic mapping of visual cortex and evaluation of the amount of orientation and scale information available in voxel representations. Similarly, a population receptive field model which encodes visual stimuli like high contrast bars into Gaussian receptive fields (Dumoulin and Wandell, 2008) with an exponential non-linearity (Kay et al., 2013) is routinely used to define retinotopic field maps (Benson et al., 2018). More complex encoding models of semantic category of visual objects (Naselaris et al., 2009; Huth et al., 2012) or language (Huth et al., 2016) have also been fit to voxel responses and examination along which dimensions of the model space the fitted weights vary the most can be used to understand the nature of what is represented.

That inverted encoding models recover the model responses, not the stimulus, is not to say that they have no useful purpose. Inverted encoding models have been fruitfully used to tease apart responses to different aspects of a compound stimulus into target and mask responses to evaluate predictions of normalization models (Brouwer and Heeger, 2011; Brouwer et al., 2015). Reconstructing model responses might be particularly important in a brain machine interface, where the model might include, for example, the response of different actuators for a robotic arm. Inverting a channel encoding model also allows for reconstruction of stimuli for which the model has never been trained, by comparing the recovered channel responses to those that would be elicited by untrained stimuli and selecting the stimulus whose channel response is most correlated with the one recovered by the inverted model (Brouwer and Heeger, 2009; Lorenc et al., 2018). Summing model receptive fields weighted by the recovered channel responses (Sprague and Serences, 2013; Sprague et al., 2014, 2016, 2018b; Samaha et al., 2016; Vo et al., 2017) is a computation similar in spirit to a vector-average read-out (Georgopoulos et al., 1986; Lee et al., 1988; Gardner et al., 2004) in that it allows each channel to “vote” for its preferred spatial location according to its reconstructed response. Thus, this approach can be viewed as a further elaboration of the inverted encoding model as it aims to determine the expected population read-out of a stimulus compatible with the measured response, rather than a model reconstruction. However, unlike the Bayesian approach (van Bergen et al., 2015; van Bergen and Jehee, 2018), it does not provide an estimate of how likely any stimulus is given the measured response. Despite these valuable usages of inverted encoding models, when the model inversion recovers theoretical channel responses such as orientation tuned channels, the properties of those channel responses should be considered a property of the model and the estimation process and not as a measurement of underlying selectivity of the hypothetical neural tuning functions (Liu et al., 2018) or the population. As a specific example, the tuning width of the channel responses should not be taken as a measure of population selectivity as it will depend on the tuning width of the particular (and arbitrary) channel basis functions used.

Our results here show that channel basis functions are only determined up to an invertible linear transform, but this does not preclude comparison of encoding models whose basis functions are not related by an invertible linear transform. In such cases, standard statistical model comparisons that take into account the number of parameters and the goodness-of-fit can be used to select the best fitting model. Because these non-linearly-relatable models make different predictions, one can also compare model predictions to other behavioral and neural measures of perceptual space to select models. As a concrete example, Brouwer and Heeger (2009) compared a six-channel hue tuning model with a four-channel cone opponency tuning model and concluded that the former was more consistent with the data in hV4. This is possible because these two models are not related by an invertible transform.

Proper inferences from computational modeling of data can only be achieved if the limits imposed by these techniques are explored and recognized by the communities that use them. Our results can be considered an example of this principle. Another analogous example to the issue that we describe here can be found in the theory and experiments of population coding of color. Indeed, the trichromatic color theory developed from the work of Young and Helmholtz (Young, 1802; Helmholtz, 1867), can only establish color matching functions up to a linear transform because they depend on the spectral power distribution of the three primary lights used in the matching experiment (Wandell, 1995). However, because the linear assumptions of color matching theory were known for over a century (Grassmann, 1854), experimenters were able to make the correct inference that the cone sensitivities in the primate retina would only need to match up to a linear transform (Baylor et al., 1987) to the color matching functions measured perceptually. Thus, the linking hypothesis between population coding in the retina and perception of colors was validated only because there was clear understanding of the limits imposed by the underlying theory.

While sophisticated new computational techniques such as inverted encoding models offer the possibility of new discovery from large and complicated datasets, they also intervene many layers of mathematical analysis between measurement and data presentation, thus creating interpretational challenges. This is not a challenge unique to human imaging, but shared with other analyses of population activity measures including electrophysiologically or through calcium imaging. Whether a computational analysis is discovering structure within data or imposing it can at times be difficult to adjudicate. For example, dimensionality reduction techniques have been used to uncover rotational dynamics in motor preparatory population activity (Churchland et al., 2012), but it could be that the computational techniques are able to extract dimensions of rotational dynamics whether or not they are in the data. One possible way to address this question is by the use of carefully designed surrogate data sets which have various components of population activity removed, to understand where effects are coming from (Elsayed and Cunningham, 2017). The larger question in assessing population stimulus representations remains as to what information is carried in a population that is not inherent in the single-unit representation. Indeed, even theoretic notions that try to decompose information into components that are represented by individual neurons and ones that are synergistically represented have difficulty in formally defining what is meant by synergistic information that arises from the population but is not in the individual units (Lizier et al., 2018). Moving forward, our analyses and understanding of population stimulus representations will need to derive from agreed on definitions for what is meant by population representations and from considerations of how much analyses impose on structure versus how much they reveal.

## Synthesis

Synthesis Statement for Author (Required):

Both expert reviewers have serious concerns of this commentary but both believe it has potential. I thus offer you a chance of revision to address their concerns. Their detailed comments are listed below.

We thank the editor for the opportunity to revise. The reviewers' comments have pushed us to further our analysis of the inverted encoding model approach and we have come to a much stronger conclusion than the previous version of the manuscript. Namely, we have now shown by mathematical derivation and simulation that the choice of channel basis functions is only determined up to an invertible linear transform [Lines 205-281]. Basically, any basis functions that are linear transforms of each other are mathematically interchangeable, such that inverting the encoding models to predict the channel responses produces a function that is only one of an infinite family of possible representations. As we now show, unimodal, bimodal or even random channel basis functions can be recovered -- and all of these are mathematically equivalent -- the choice of presenting one or the other is completely arbitrary. We believe that this is a much stronger point for the commentary to make that will be of value to the community of neuroscientists using this technique. We want to thank the reviewers for pushing us to make a sharper point.

We would also like to emphasize that the critique we are making is about the inverted encoding model and not about modeling in general. Both reviewers bring up points related to model fitting issues which we are in complete agreement with. However, our critique is not with fitting models to BOLD data, but with the unique interpretational issues that come from trying to invert that process. Since we now show that the model used is only constrained up to a linear transform, this inversion step is what we argue to be a poor way to evaluate population representations. Basically, recovering a unimodal function from BOLD data, when this function is mathematically equivalent to a bimodal or even random function, is not displaying anything inherent in the structure of the data and is therefore misleading. Approaches that we highlight such as the Bayesian method do not rely on this intermediate stage of the analysis, but provide a posterior function that represents how much is known about the stimulus from the neural response, something more similar to a traditional tuning curve and this is the reason we advocate for it. We have edited throughout the manuscript to make our point more clear (e.g., see Lines 345-395). As a result, we feel our commentary is stronger and more focused than before.

Below we provide a point-by-point response to reviewers' comments. We have highlighted the most major changes in the manuscript with a green font per journal requirement.

Reviewer 1

This commentary makes the observation that inverted encoding models (broadly, fitting parameters of an underlying model to observed data via by direct matrix inversion) recovers a model of the underlying mechanism that is dependent on the initial assumptions. I'm not sure that this is quite as novel an observation as the authors claim - nor that the alternative they propose (recovering stimuli from population responses) is as useful or as insightful as we might hope.

Indeed, we agree with the reviewer's general observation that model inversion depends on the initial assumptions. However, our observation is that many practitioners using this approach are seemingly unaware of this fundamental limit. For example, the suggestion that the recovered channel response function is a measure of population-level stimulus representation ignores the fact that the form/shape of these functions depends on the initial model assumptions. That is precisely what we want to comment on and bring awareness to. In this revision, we have obtained new derivations and simulations showing that the channel basis functions are only determined up to an invertible linear transform (see above), which has significantly strengthened our observation. Furthermore, we also demonstrate that the alternative method of reconstructing the stimulus is much less sensitive to model assumptions, thus making it a better method.

The observation (that model fits recover things that depend on the underlying model) seems true but also rather trivial. The authors claim at some point that inverted encoding models are essentially fancy forms of linear regression and the point is instructive: we are warned in Statistics 101 that linear regression is only appropriate when the underlying data distribution is, in fact, linearily dependent on the parameters. As far as I can tell, this paper is reiterating the same advice: if the underlying model is inaccurate, you will recover misleading parameters. The authors illustrate this nicely with the bimodal vs unimodal distribution example in their simulation and the observation from their recent JNS paper that SNR can affect fit estimates is an important point that should, I think be more widely understood. But the presence of complexities in model fitting does not, I think, invalidate the entire enterprise.

We appreciate this comment. Indeed, our results can be construed as a case of mis-specified model giving rise to misleading results. However, as stated above, this point is not obvious to practitioners in the field using the inverted encoding model. Moreover, our new derivations and simulations show that the bimodal model is not, technically speaking, mis-specified as it is a linear transform away from the unimodal model. This also means that examining goodness-of-fit is not effective for assessing the appropriateness of models (the goodness-of-fit of the unimodal and bimodal models are mathematically equally), if that is what the reviewer implied regarding Statistics 101. The problem is much more pernicious. Finally, we want to clarify that our commentary is not aimed at invalidating the enterprise of model fitting, rather at pointing out that *inverting* the encoding model produces results that cannot be meaningfully used to assess population tuning. We have refined our discussions on these points [Lines 329-385].

They also mention a widely-used class of modelling procedure that recovers the parameters of the underlying model without direct matrix inversion (e.g. Dumoulin et al 2008). This is simply a more general approach to model fitting that is able to cope with nonlinearities in the data at the expense of some additional computational complexity. It seems worth emphasising this point more strongly.

We agree about the advantages of the model fitting approach of Dumoulin et al which we discuss in the text specifically [Lines 355-357]. We also point out [Lines 345-360] that the Dumoulin approach is fundamentally different from the inverted encoding model approach. The Dumoulin approach examines the fit parameters for each voxel to determine the receptive field location and size. The inverted encoding model approach does not examine the fit parameters to determine the selectivity of voxels, but instead reconstructs the (arbitrary up to a linear transform) channel responses.

This commentary is a warning to people to check their model assumptions and/or be wary of straightforward matrix inversion for estimating model parameters. But in a way I think it strengthens rather than weakens the arguments for encoding models in general. In the end, these models allow us to test different hypotheses about the underlying neuronal representation. An example (mentioned by the authors) is Brouwer and Heeger's work in the late 2000s where they explicity test models of opponent color channels vs a more distributed representation of color across visual areas. This approach has value precisely because Brouwer and Heeger are testing the underlying assumptions instead of attempting to reconstruct the input stimuli. Incidentally, the elegance and rigor of these color papers contradict the authors' use of chromatic coding as a 'simple' stimulus representation that is trivial to understand (L301).

We agree that model comparison is fundamentally important when different models can be mathematically distinguished and now explicit discuss this and the approach of Brouwer & Heeger [Lines 386-395]. We would also highlight that our critique is not that matrix inversion for estimating model parameters is problematic. Our critique is that the inverted encoding model, as typically used, simply reconstructs the intermediate state of the model from the BOLD rather than compares predictions of different models. Brouwer & Heeger use PCA analysis of the voxel responses (and later, estimated channel responses) to determine qualitatively whether the color representation in different visual areas matches the hue-encoding model (which shows a systematic progression of colors in the first two PCA component space) or the cone-opponency model (which does not show a systematic progression of colors in its PCA space). This is possible because the two models are not linear transforms of each other and because the models predictions are explicitly tested. Our manuscript now makes explicit when it is mathematically impossible to compare even seemingly different models such as unimodal and bimodal, which we believe to be an important and previously unappreciated limitation of the approach. Also, we agree that our previous description of chromatic coding did not do justice to the elegance and rigor of color perceptual neuroscience and have therefore exchanged the description with one that links more directly with our findings [Line 392-430].

Finally, the Bayesian approach to stimulus reconstruction is complementary rather than an alternative to encoding models. Mind reading is fun. But the goal of many of the researchers who generate encoding models is to interrogate the underlying stimulus representation in cortex through an iterative process of model generation, testing and refinement based on, among other things, electrophysiological data. The warning here is that assumptions of model linearity might trip you up sometimes and this warning might be worth repeating. Certainly, it is more important when we are examining cortical regions where electrophysiology provides incomplete guidance about the underlying neuronal representations (L244). However, I am also sure that not modelling at all is a bad idea and it seems at first glance that this is what the authors are suggesting. Perhaps the commentary could be re-written to identify the dangers inherent in fitting a single, incorrect linear model while providing more guidance on how to do model fitting well?

We completely agree. There seems to be some misunderstanding here because we certainly do not want to suggest modeling is bad. Indeed, the Bayesian approach we advocated is built upon the encoding model. Rather, our critique is about the *inversion* step of inverted encoding models which aims not to test or compare different models, but to produce a visual output of the analysis to summarize population representation. Because this inversion step is only determined up to a linear transform it is an ambiguous and problematic representation. The Bayesian approach does not suffer from this problem -- instead it gives a representation of what can be determined about the stimulus from the population of activity. We have further clarified this point throughout the revised text.

Reviewer 2

This manuscript is a response to the commentary “Inverted encoding models assay population-level stimulus representation, not single-unit neural tuning” (Sprague et al., 2018). Using a procedure identical to those reported by Liu et al.(2018), the authors demonstrated that SNR affects the shape of the reconstructed channel responses. The simulation result points out the importance of SNR in interpreting the model outcome. However, the example reported was an extreme case, which does not weaken the validity of the inverted encoding model with adequate model specifications in showing the population-level stimulus representation. Generally, the pitfalls of the inverted encoding model were over-claimed.

We appreciate this comment, which made us to further examine the foundation of the inverted encoding model. We now demonstrate that inverted encoding model is only specified up to a linear transform (see introduction paragraphs of this letter). Thus, the bimodal channel basis functions are not an extreme case, but a mathematically equivalent model to the more typical unimodal channel basis functions. We believe this new observation substantially weakens the validity of the inverted encoding model because it is mathematically impossible to determine that the bimodal or even random channels are inadequately specified models.

1. The authors reviewed different units of ordinates used in the channel response profile and discussed this as “a rather odd feature". I don't agree with this. The ordinate of the channel response was usually labeled the same as the unit of BOLD signal change in previous studies. It is reasonable to have the ordinate of the predictor in the same unit as the dependent variable, given that the weights are coefficients without units. As the BOLD responses can be defined as the percent signal change, the beta value from a general linear model, the z-score, etc., the unit of the ordinate varies accordingly.

Also, the author claimed that “the units of the ordinate are arbitrary in the sense that they can be manipulated by simply changing the maximum response of the modeled channels” and suggested that this can be avoided by plotting the ordinate in a percentage of the full model response. Is this necessary? Because the inverted encoding model is generally set up with basis functions defined as sinusoidal functions, the maximum response of the modeled channels is already fixed at 1.

The following analogy was confusing: “if one were fitting a linear regression to predict age from percent signal change, the ordinate would be in units of what is being predicted - years of age - rather than in the units of the predictor - percent signal change.” In the inverted encoding mode, the BOLD response is predicted by the channel responses. However, the authors discussed the BOLD response as the predictor in this example.

The reviewer is confusing encoding models with *inverted* encoding models. In particular, the statement above “In the inverted encoding model, the BOLD response is predicted by the channel responses” is incorrect. That is a description of an encoding model. The inverted encoding model is the opposite--the BOLD responses are used to predict the channel responses. Specifically, BOLD responses are assumed to be weighted averages of the channel responses and this holds true for both training data (B1=WC1) and test data (B2=WC2). The inverted encoding approach uses the training data (B1) to estimate W and then use the estimated W to compute C2 (channel response). Thus, it is predicting the channel response (unit-less), rather than the BOLD response. As this is the central point of the commentary, we have edited the section to make the distinction more clear [Lines 135-169]. In addition, the newly added mathematical derivations should make it quite clear what the inverted encoding model is predicting [Lines 205-281].

2. The current manuscript considers “an extreme case of poor model specification in which channels are assumed to have a bimodal shape, instead of a unimodal shape". However, the inverted model was usually set up with unimodal basis functions in accordance with the electrophysiological evidence. It would be valuable to run simulations based on more practical cases using the same synthetic data. For example, can the inverted encoding model reconstruct the channel response for plaid motion? When stimulated with overlapping gratings moving in two directions, it is reasonable for the hypothetical channel response to be bimodal, with unimodal tuning functions. Does the Bayesian approach generate a bimodal distribution in this case?

In the new version, we are able to make a much stronger point than previously. It is no longer the case that the bimodal channel basis functions are an extreme case of poor model specification. The bimodal channel basis functions are an invertible linear transform of the unimodal basis functions and (as we now show) this means that they are mathematically interchangeable. As for plaid motion, it is well known that cortical responses can show non-linear combination, a full account for these plaid vs. component effects in the context of channel encoding models would require additional consideration of normalization and other non-linear effects with proper model comparisons. This is potentially an interesting direction to consider, but is beyond the scope of the current manuscript which is aimed at pointing out that inverting encoding models recovers a function that is only specified up to an invertible linear transform.

3. What are the results of these two models when the channels were assumed in typical unimodal shape? What are the goodness-of-fit under the correct (unimodal) and the incorrect (bimodal) basis functions?

As we have now added to the text, the goodness-of-fit for the two channel basis functions are identical because they are related by an invertible transform [Lines 205-262]. We believe this point to be crucial because it means that there is no possibility to do model comparison statistics to ask which is the better model -- the two models (and in fact an infinite family of models) are mathematically equivalent.

4. As to me, the highlight of this commentary is that SNR affects the shape of the response profile -- change from bimodal to unimodal. Does such an effect only exist in this extreme case? It has been found that SNR affects the bandwidth of the channel response with unimodal basis function. Does SNR affect any other properties of the channel response with typical unimodal basis function?

In general, as SNR lowers, the inverted encoding model will recover flat functions and as SNR increases the recovered functions will become closer to the assumed channel basis function in the analysis. As these channel basis functions cannot be distinguished if they are linear transforms of each other, it is possible to produce a wide range of behavior dependent on the arbitrary choice of channel basis functions. This is the reason why we are critiquing the inverted encoding model approach -- it can make pretty much any picture out of the data that you would like it to by the arbitrary choice of channel basis functions.

Minor issues:

Abstract:

L15 “the inverted encoding model is extended...". The description is not clear. There should be a brief summary of the Bayesian approach.

We feel it would be rather unwieldy to explain the Bayesian approach in the Abstract. The important point is that this approach reconstructs the stimulus, which was stated. The technical details are less important and would also take too much text to explain in an Abstract. The main text contains a full explanation of the Bayesian approach.

L140 “forty-two” Why this particular number?

This is just our way to say that it is arbitrary. Because the inverted encoding model reconstructs the model (C2 in the previous point), the magnitude on these channel responses depend on the magnitude of the channel basis functions, which is unitless and arbitrary.
